# Gene flow signature in the *S*-allele region of cultivated buckwheat

**DOI:** 10.1186/s12870-019-1730-1

**Published:** 2019-04-03

**Authors:** Nobuyuki Mizuno, Yasuo Yasui

**Affiliations:** Graduate School of Agriculture, Kyoto University, Sakyo-ku, Kyoto, 606-8502 Japan

**Keywords:** Buckwheat, Crop evolution, GBS, Heteromorphic self-incompatibility

## Abstract

**Background:**

Buckwheat (*Fagopyrum esculentum* Moench.) is an annual crop that originated in southern China. The nutritious seeds are used in cooking much like cereal grains. Buckwheat is an outcrossing species with heteromorphic self-incompatibility due to its dimorphic (i.e., short- and long-styled) flowers and intra-morph infertility. The floral morphology and intra-morph incompatibility are both determined by a single *S* locus. Plants with short-styled flowers are heterozygous (*S/s*) and plants with long-styled flowers are homozygous recessive (*s/s*) at this locus, and the *S/S* genotype is not found. Recently, we built a draft genome assembly of buckwheat and identified the 5.4-Mb-long *S*-allele region harbored by short-styled plants.

In this study, the first report on the genome-wide diversity of buckwheat, we used a genotyping-by-sequencing (GBS) dataset to evaluate the genome-wide nucleotide diversity within cultivated buckwheat landraces worldwide. We also investigated the utility of the *S*-allele region for phylogenetic analysis of buckwheat.

**Results:**

Buckwheat showed high nucleotide diversity (0.0065), comparable to that of other outcrossing plants, based on a genome-wide simple nucleotide polymorphism (SNP) analysis. Phylogenetic analyses based on genome-wide SNPs showed that cultivated buckwheat comprises two groups, Asian and European, and revealed lower nucleotide diversity in the European group (0.0055) and low differentiation between the Asian and European groups.

The nucleotide diversity (0.0039) estimated from SNPs in the *S*-allele region is lower than that in genome-wide SNPs. Phylogenetic analysis based on this region detected three diverged groups, S-1, S-2, and S-3.

**Conclusion:**

The SNPs detected using the GBS dataset were effective for elucidating the evolutionary history of buckwheat, and led to the following conclusions: (1) the low nucleotide diversity of the entire genome in the European group and low differentiation between the Asian and European groups suggested genetic bottlenecks associated with dispersion from Asia to Europe, and/or recent intensified cultivation and selection in Europe; and (2) the high diversification in the *S*-allele region was indicative of gene flows from wild to cultivated buckwheat, suggesting that cultivated buckwheat may have multiple origins.

**Electronic supplementary material:**

The online version of this article (10.1186/s12870-019-1730-1) contains supplementary material, which is available to authorized users.

## Background

Buckwheat (*Fagopyrum esculentum* Moench.; 2*n* = 2*x* = 16), a member of the Polygonaceae family, is an annual crop that originated in southern China [1, 2]. The seeds (strictly achenes) are used as cereal grains in the same way as rice (*Oryza sativa*) and wheat (*Triticum aestivum*); because buckwheat does not belong to the Poaceae family, it is often referred to as a pseudo-cereal. Buckwheat has excellent cultivation properties, with a short growing period and tolerance of cool climate and high elevation. Buckwheat is therefore widely cultivated in temperate zones throughout Eurasia and is used in many traditional foods, such as soba (Japanese noodles), memil guksu (Korean noodles), pizzoccheri (Italian pasta), and galettes (French pancakes). Buckwheat seeds are dense in starch and high-quality protein with a well-balanced amino acid composition [3] and are an important source of dietary fiber, trace elements, and phenolic compounds [4, 5]. Because of its high nutrient content and lack of gluten, buckwheat is now widely cultivated in regions beyond Eurasia, including in the USA, Canada, Australia, and New Zealand.

To identify agronomically useful genes, buckwheat linkage maps have been constructed using various molecular markers, including isozyme variations [6], simple sequence repeats (SSRs) [7], and amplified fragment-length polymorphisms (AFLPs) [8]. Recently, a microarray marker system and a genome-wide linkage map for common buckwheat were developed [9]. They later confirmed that genomic selection using genome-wide microarray markers was an effective approach for improving buckwheat yield [10]. In addition, our research group constructed the Buckwheat Genome DataBase (BGDB) [11]. Using BGDB, various agronomically useful genes, such as those controlling flavonoid biosynthesis and encoding 2S albumin-type allergens and granule-bound starch synthases (GBSSs), have been identified [11–14]. Thus, the genetic tools for buckwheat breeding are highly developed.

Evaluating genetic diversity is a crucial step for exploring agronomically useful genes in crop species. Buckwheat is an outcrossing species with heteromorphic self-incompatibility (SI) due to its dimorphic flower types (i.e., short- and long-styled flowers), each incompatible with flowers of the same morph, but compatible across morphs [15, 16]. The floral morphology and intra-morph incompatibility are both determined by a single genetic locus, *S*. Plants with short-styled flowers are heterozygous (*S/s*) and those with long-styled flowers are homozygous recessive (*s/s*) at this locus [15, 17]. Thus, like other outcrossing crops such as maize (*Zea mays*), buckwheat is expected to maintain substantial intraspecies diversity. Indeed, isozyme analysis indicates that buckwheat has great genetic diversity, comparable to that of outcrossing wild plant species [18]; the heterozygosity of cultivated populations is higher than that seen in wild populations of the ancestral species, *F. esculentum* ssp. *ancestrale*, which is found in southern China. Subsequent AFLP and SSR analyses suggested that *F. esculentum* ssp. *ancestrale* is composed of two distantly related phylogenetic groups, the Tibetan and the Yunnan-Sichuan groups, and that cultivated buckwheat landraces belong to the Tibetan group [1, 19]. However, these analyses were based only on similarity of DNA banding patterns, and the average number of nucleotide differences per nucleotide site (i.e., the nucleotide diversity) [20] was not estimated. To date, no information on genome-wide nucleotide diversity within *F. esculentum* is available. Thus, there is great interest in investigating the nucleotide diversity of *F. esculentum* using newer and superior genomic tools.

Recently, we built a draft genome assembly of buckwheat and then applied genotyping-by-sequencing technology (GBS) [21] to a group of buckwheat landraces from around the world [11]. The GBS method, which uses sequences of amplified genomic DNA fragmented by restriction enzymes, has become increasingly popular for detecting large numbers of SNPs [22]. We used the draft genome assembly as reference for GBS markers, and successfully identified the *S-*allele region, which consisted of 332 scaffolds encompassing 5.4 Mbp [11]. The region contains sites at which GBS reads were mapped in short-styled plants but not in long-styled plants, and harbors two *S*-allele-specific genes, *S-ELF3* and *SSG2*, that exist only in the genomes of short-styled plants. The genotypes of short-styled and long-styled plants are thus hemizygous and null homozygous, respectively [15]. This is similar to the situation for human sex chromosome genes; the hemizygous state of Y-chromosome genes has proved a useful feature for clarifying human phylogenetic structure [23]. Thus, we predicted that comparisons of DNA sequences in the *S*-allele region would be a powerful tool for elucidating phylogenetic relationships among buckwheat landraces.

In this study, we evaluated the genome-wide nucleotide diversity within worldwide landraces of cultivated buckwheat using the published GBS data. This is the first report of genome-wide nucleotide diversity within *F. esculentum*. We discuss the utility of the *S*-allele region for phylogenetic analysis of the buckwheat species, and demonstrate possible gene flows from wild to cultivated buckwheat deduced from the diversified *S*-allele region.

## Results

### Overall SNP detection and nucleotide diversity

We obtained an average of 7.2 million reads per plant (corresponding to 726.1 Mbp). After filtering the sites detected by GBS for 46 buckwheat plants, we retained 7,154,454 sites, corresponding to 0.61% of the reference genome, for further analysis (Table 1). From the 7,154,454 sites, we detected 255,517 SNP sites, representing a SNP density of 0.036 (one SNP per 28 bp). We compared the number of mapping sites, the SNP density, and the nucleotide diversity between short- and long-styled plants and found no significant differences (Table 1).

**Table 1:** Comparisons of nucleotide diversity

Category^a^	Scaffolds used as reference^b^	Number of samples	Number of sites retained after mapping	Number of SNPs	SNP density	Mean genetic diversity (pi)
All	All scaffolds	46	7,154,454	255,517	0.036	0.0065
Asian	All scaffolds	32	7,191,709	244,709	0.034	0.0065
European	All scaffolds	14	7,314,616	136,369	0.019	0.0055
Short-styled plant	All scaffolds	23	6,986,824	208,928	0.030	0.0065
Long-styled plant	All scaffolds	23	7,104,722	207,568	0.029	0.0064
Short-styled plant	*S-*allelic scaffolds	23	60,108	1123	0.019	0.0039

^a^All, containing all samples; Asian, containing samples from Asian countries; European, containing samples from European countries

^b^All scaffolds, all 387,594 scaffolds in Fes_r1.0; S-linked scaffolds, 332 of *S*-linked scaffolds obtained by Yasui et al. (2016) [11]

### Population structure and phylogenetic analysis

**Fig. 1:**
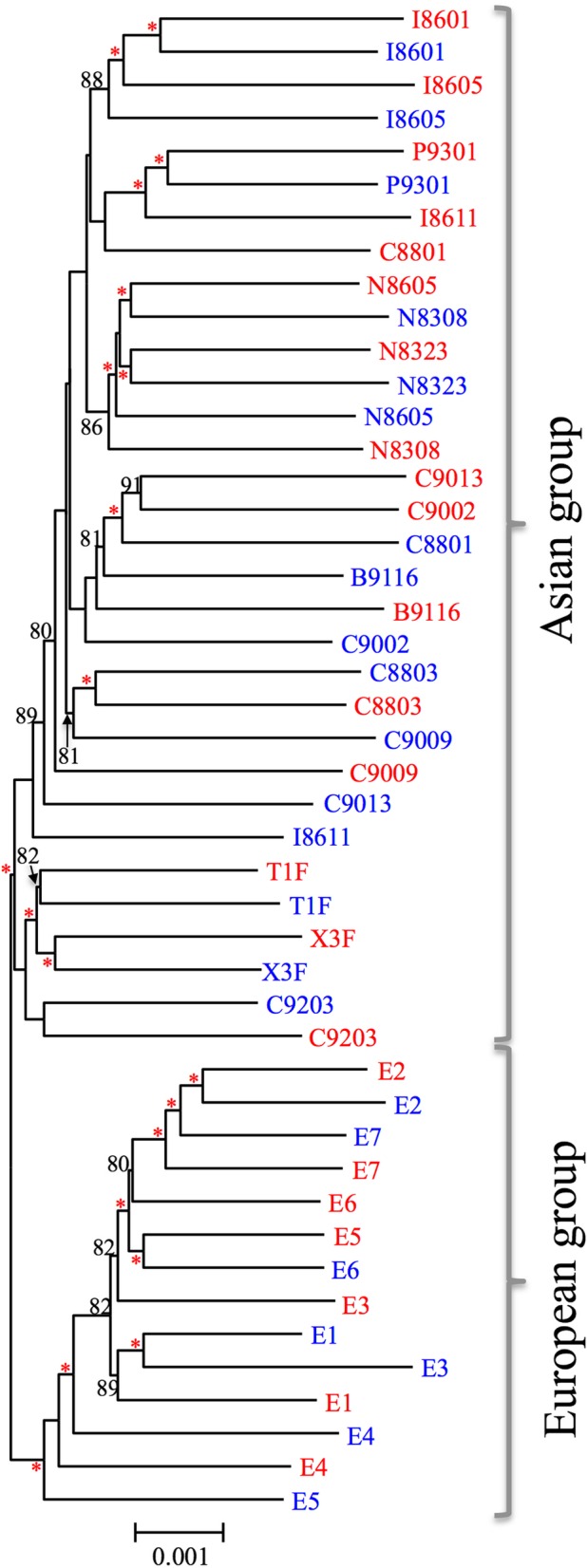
Neighbor-joining (NJ) tree of 46 cultivars of common buckwheat based on 7.15 Mbp including 255,517 SNPs. Red and blue represent short- and long-styled plants, respectively. Numbers above branches show bootstrap values based on 100 replicates (those less than 80% are not shown) and red asterisks indicate bootstrap values of 95% or over. The scale bar corresponds to 0.001 substitutions per nucleotide site

**Fig. 2:**
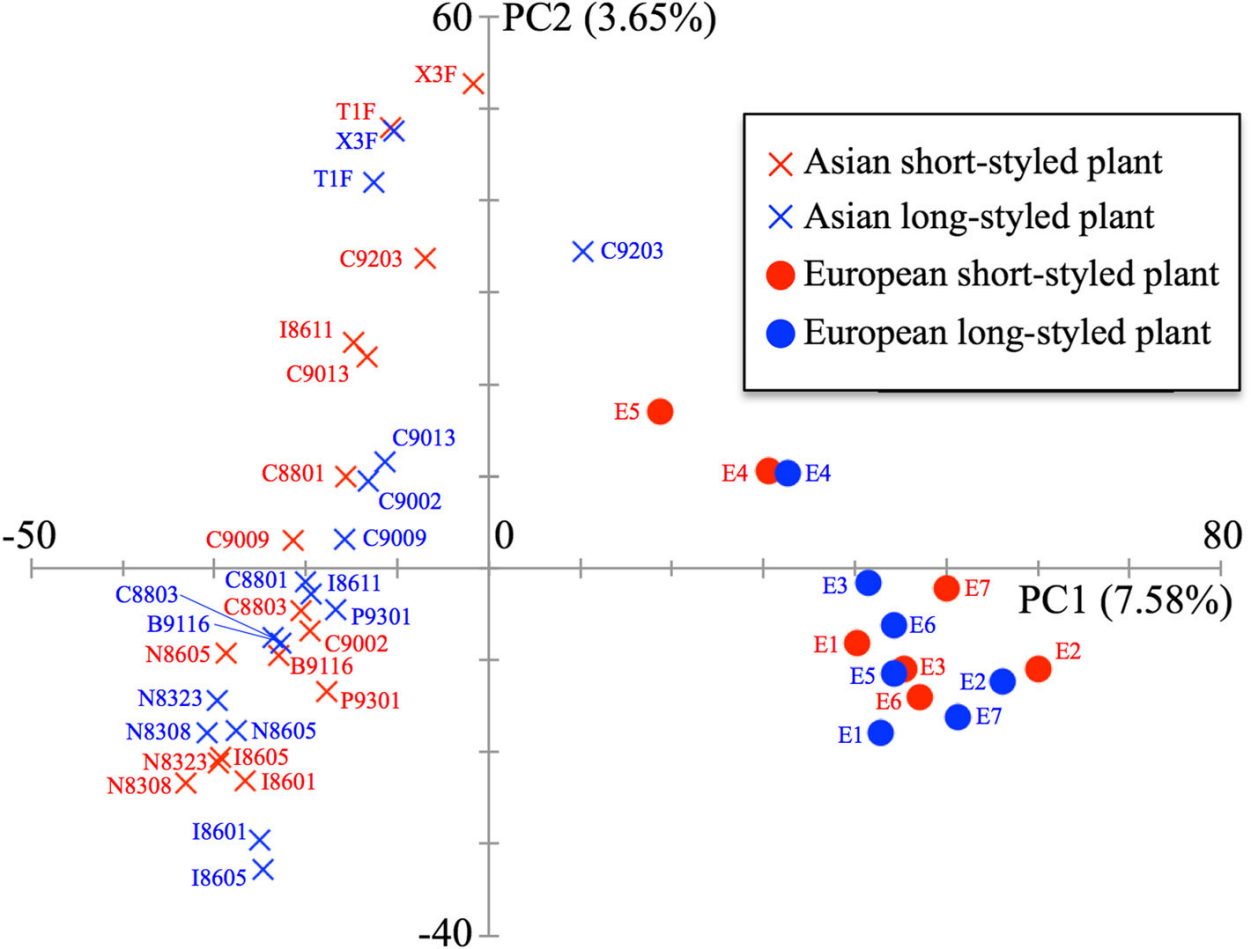
Principal-component analysis (PCA) of 46 cultivars of common buckwheat based on GBS data mapped on all genome scaffolds. Graph of the first two axes (*x-*axis for PC1 and *y-*axis for PC2) from PCA. The proportion of variance explained by each component is given in parentheses along each axis. Red and blue represent short- and long-styled plants, respectively

We calculated genetic distances among common buckwheat landraces and constructed a phylogenetic tree by the NJ method using 7,154,454 sites including 255,517 SNP sites. The NJ tree showed that 46 cultivars were largely divided into two groups, Asian (32 cultivars) and European (14 cultivars) (Fig. 1). The phylogenetic relationships among cultivated buckwheat were well associated with geographic distribution; samples from one country were phylogenetically closely related in the NJ tree (e.g., I8601 and I 8605 for India, N8323 and N8605 for Nepal, and T1F and X1F for Japan). The PCA plot also showed that 46 cultivars were largely divided into two groups composed of Asian and European cultivars, respectively (Fig. 2).

To clarify the population structure of buckwheat, we subjected 255,517 SNPs segregated in 46 GBS to ADMIXTURE analysis. Cross-validation error was lowest at *K* = 1 and gradually increased as *K* increased (Additional file 1: Figure S1). At *K* = 2, the cross-validation error was also low, indicating that the optimal number of ancestral populations was *K* = 1 or *K* = 2. Although the lowest cross-validation error was actually observed at *K* = 1, the NJ tree and PCA implied that there were two groups (one including only Asian landraces and the other only European landraces), indicating that *K* = 2 is suitable for grouping the 46 common buckwheat landraces. We refer to the two groups hereafter as the Asian and European groups.

To compare nucleotide diversity between the Asian and European groups, we identified SNPs and calculated the genome-wide mean nucleotide diversity of each group (Table 1). The SNP density was much lower in the European group (0.019) than in the Asian group (0.034). Nucleotide diversity was also lower in the European (0.0055) than in the Asian (0.065) group. However, even though the NJ tree and PCA plot indicated that there were two groups, the Fst value between Asian and European cultivars was not very high (Fst = 0.068).

### Diversity of S-linked scaffolds

We previously identified the *S*-allele region, consisting of 332 scaffolds encompassing 5,393,196 bp, to which short-style-specific sites were mapped [11]. To analyze the nucleotide diversity of this region, we identified SNPs on these 332 scaffolds in 23 short-styled plants. From the 60,108 sites (0.11% of the total length of the 332 scaffolds), we detected 1123 SNPs. The SNP density (0.019) and genetic diversity (0.0039) in the *S*-allele scaffolds were lower than those in all genomic scaffolds (Table 1).

**Fig. 3:**
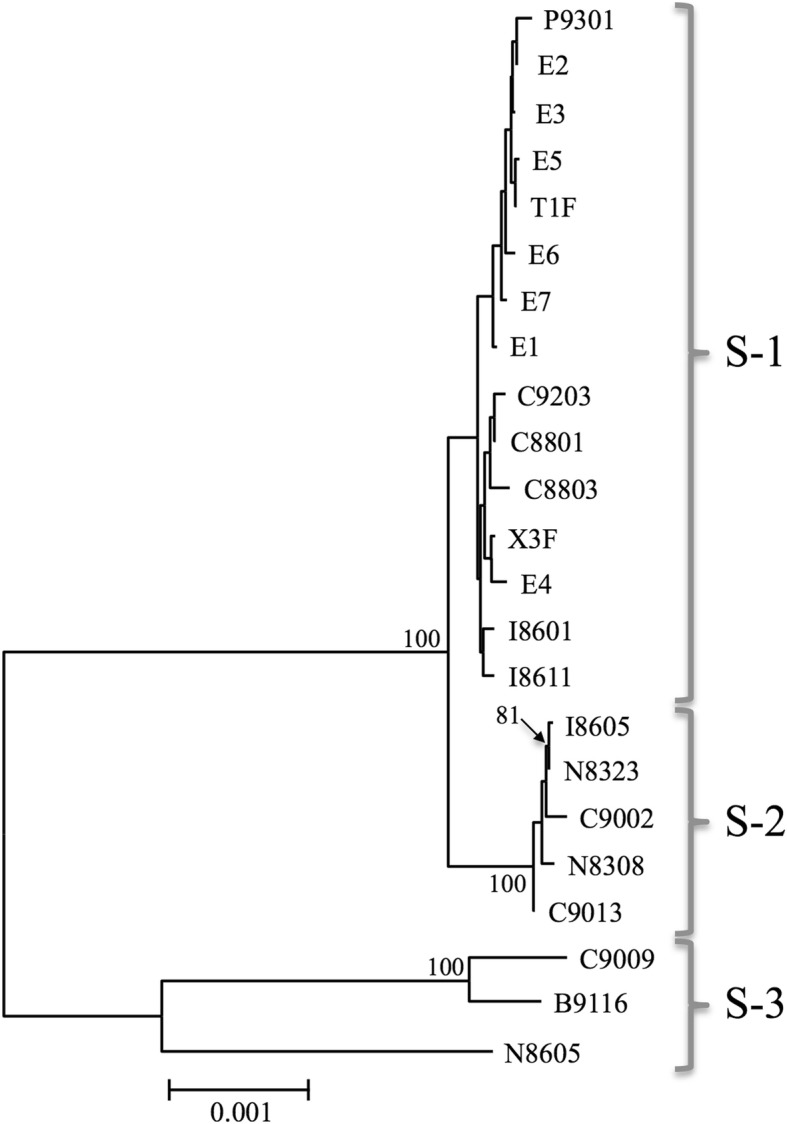
Neighbor-joining (NJ) tree based on GBS sequences (60.1 Kbp) mapped on 332 *S-*allelic scaffolds. All 23 samples are short-styled plants harboring an *S* allele (genotype, *S/s*). Numbers above branches show bootstrap values based on 100 replicates (those less than 80% were not shown). The scale bar corresponds to 0.001 substitutions per nucleotide site

To uncover the phylogenetic relationship among the *S*-allele region obtained from 23 short-styled plants, we constructed an NJ tree based on the *S*-allele region using 60,108 sites, including 1123 SNP sites. The NJ tree based on the *S*-allele region showed that common buckwheat plants of the short-styled phenotype (*S/s* genotype) were largely divided into three groups, which we named S-1, S-2, and S-3 (Fig. 3). The phylogenetic relationship identified on the basis of the *S*-linked scaffolds was identical to that implied by analysis of *S-ELF* and *SSG2* [15] but different from that of the NJ tree based on genome-wide SNPs (Fig. 1). The S-1, S-2, and S-3 phylogenetic groups were composed of fifteen, five, and three cultivars, respectively. These groups were also detected by the PCA (Additional file 2: Figure S2). The nucleotide diversity of the *S*-allele region (0.0039) was lower than that of all scaffolds (Table 1). The average nucleotide distance and Fst value between the phylogenetic groups in the *S*-linked scaffolds were high (Table 2). In particular, S-3 was clearly more distantly related to S-1 and S-2 than they were to each other.

**Table 2:** Pairwise comparisons of the average genetic distances and Fst values between pairs of three phylogenetic groups

Gropups^a^	S-1	S-2	S-3
S-1		0.00047	0.00656
S-2	0.281		0.00669
S-3	0.640	0.625	

Above diagonal, average genetic distance; below diagonal, Fst

^a^S-1, S-2, and S-3 are phylogenetic groups detected by NJ analysis (Fig. 3) and PCA (Additional file 2: Figure S2)

## Discussion

### Genome-wide and S-allele-region-specific nucleotide diversity in common buckwheat

In this study, we obtained 255,517 genome-wide SNPs using the GBS method, which is capable of producing larger numbers of genome-wide SNPs than had been obtained in previous studies of this species. Furthermore, the published genome sequence [11] enabled us to estimate the nucleotide diversity, which revealed that the nucleotide diversity of common buckwheat (0.0065) was comparable to that of other outcrossing plants, such as maize (*Zea mays*, 0.0064) [24] and sunflower (*Helianthus annuus*; 0.0056) [25], and higher than that of selfing crops such as rice (*Oryza sativa*, 0.0024) [26] and soybean (*Glycine max*; 0.0019) [27]. This higher nucleotide diversity is likely due to buckwheat’s outcrossing mating system and/or to gene flow from wild buckwheat, *F. esculentum* ssp. *ancestrale*.

The genetic basis of the heteromorphic SI system is similar to that of sex chromosomes in the plant and animal kingdoms. There have been numerous reports on the nucleotide diversities of sex chromosomes [28–31]. In humans, for example, the ratios of the nucleotide diversities of the X and Y chromosomes to those of the autosomal chromosomes are 0.62 and 0.20, respectively [32]. The lower nucleotide diversities observed in sex chromosomes are an expected result of the lower effective population size: the effective population sizes of the human X and Y chromosomes correspond to 3/4 and 1/4 that of autosomal chromosomes, respectively. In this study, we confirmed that the nucleotide diversity of the *S*-allele region is similarly lower than that of the genome as a whole: the ratio of the nucleotide diversity of the *S*-allele region to that of the whole genome is 0.62. That this number is much higher than the expected value (0.25) is probably due to the low density of functional genes in the *S*-allele region [11] and/or to recent multiple gene flows, as discussed in the following section. Much higher genetic diversity of *S*-allelic region than expected also indicates that *S*-allelic region is not under purifying selection.

### Gene flow inferred from S-allele divergence

The phylogenetic relationships deduced from the 332 *S*-allele scaffolds (Fig. 3) were not congruent with those based on genome-wide SNPs (Fig. 1): three widely diverged phylogenetic groups, S-1, S-2, and S-3, were detected in the data shown in Fig. 3 (from the *S*-allele region) but not in Fig. 1 (from the whole genome). It is noteworthy that the three phylogenetic groups of *S*-allele regions are well diverged. In particular, S-3 is strongly divergent from the other two groups: the nucleotide diversity between S-3 and the other two groups is around 0.006 (Table 2). Using a rough molecular clock rate of 0.01 synonymous nucleotide substitutions per million years (e.g., 0.011 for *Gossypium* species and 0.016 for *Arachis* species) [33, 34], this would indicate that group S-3 diverged from the other groups 0.3 million years ago. The earliest plausibly identified buckwheat archeological pollen specimen, found in northern China, has been dated to 5000 to 6000 BP [35]. Thus, it is unlikely that mutations detected in *S*-alleles derive from the origin of buckwheat cultivation. Considering the possibility of cross-compatibility resulting in fertile hybrids [36] and the overlap in habitats [19] between cultivated and wild buckwheat, we concluded that the diverged *S*-allele sequences were introgressed from wild buckwheat, *F. esculentum* ssp. *ancestrale*, and that the introgressions between wild and cultivated buckwheat are also attributable to high genome-wide diversity.

### S-allele region as a phylogenetic tool for elucidating the origin and diffusion of cultivated buckwheat

ADMIXTURE and phylogenetic analyses and PCA classified 46 buckwheat cultivars into two groups, the Asian and European groups, with a low Fst value (Fst = 0.068). The European group exhibited lower nucleotide diversity than the Asian group (Table 1). From archeological studies of the pollen and macrofossil (i.e., charred seeds) records, it has been suggested that buckwheat was introduced into Europe during the period 4000–2800 BP, though it did not become a popular crop until the Late Medieval period [37]. Both the lower nucleotide diversity in the European group and the weak differentiation between the Asian and European groups are likely due to genetic bottlenecks associated with dispersion from Asia to Europe, and/or the recent intensified cultivation and selection. The low genetic variation and loss of alleles detected by isozyme analysis also support this hypothesis [38].

Y chromosomes, which retain sequential records of the accumulation of nucleotide diversity, have been used to detect ancestral haplotypes and to trace human migrations [39]. Although genome-wide SNPs have illuminated the population structure and population differentiation of cultivated buckwheat, the SNPs in *S*-linked scaffolds may shed light on different aspects of the diffusion history of the species. In this study, we detected three major *S*-allele groups, S-1, S-2, and S-3 (Fig. 3). The phylogenetic relationship implied by *S*-linked scaffolds suggested the introgressions from wild relatives as discussed above. This is due to the suppression of recombination in *S*-allelic region like sex chromosomes. Thus, phylogenetic analysis using *S*-allelic region is expected to offer better understanding of the origin and global diffusion of cultivated buckwheat.

In this study, we did not find type S-1 *S*-allele sequences in the populations from Nepal and Bhutan. Based on our identification of the three diverged *S*-allele groups, the possibility that cultivated buckwheat had multiple origins should also be considered. Considering that remains of buckwheat seeds appeared in west-central Nepal from 3000 BP [40], we should consider the possibility that buckwheat was independently domesticated around the Himalayan region, including Nepal, Bhutan, and southwestern China. To elucidate the origin and global diffusion history of cultivated buckwheat, we would need to expand our sample set to include many more samples of cultivated buckwheat from around the world, as well as samples of wild buckwheat, *F. esculentum* ssp. *ancestrale*, from China.

## Conclusion

Based on genome-wide SNPs obtained using GBS technology, we successfully estimated the nucleotide diversity (0.0065) of buckwheat, which is comparable to that of other outcrossing plants, such as maize and sunflower. Phylogenetic analyses based on genome-wide SNPs also showed that cultivated buckwheat is composed of two groups, Asian and European. The low nucleotide diversity of the European group and the low differentiation between the Asian and European groups are consistent with genetic bottlenecks associated with dispersion from Asia to Europe, and/or the recent intensified cultivation and selection of buckwheat in Europe. These results based on genome-wide SNPs are congruent with those of previous studies based on isozyme variation. The nucleotide diversity (0.0039) estimated using SNPs in the *S*-allele region was lower than that estimated using genome-wide SNPs, reflecting the smaller population size of the *S*-allele region as compared to the genome as a whole. The data also indicated the likelihood of gene flow from wild to cultivated buckwheat and the possibility of multiple origins for cultivated buckwheat. In conclusion, phylogenetic analysis using the *S*-allele region can offer a better understanding of the origin of cultivation and the global diffusion history of cultivated buckwheat.

## Methods

### Genotype-by-sequencing data and SNP detection

We used published GBS reads obtained using *EcoR*I and *Mse*I restriction enzymes (DRA accession number DRA004489, ftp://ftp.ddbj.nig.ac.jp/ddbj_database/dra/fastq/DRA004/DRA004489) [11]. The GBS dataset represented 23 short-styled and 23 long-styled buckwheat landraces originating from a wide range of locations within Eurasia (Additional file 3: Table S1). Low-quality reads and adaptors were trimmed using Trimmomatic-0.36 [41] with the options SLIDINGWINDOW:5:25 and MINLEN:40. The adaptor sequences used were CACGACGCTCTTCCGATCT and ACCGCTCTTCCGATCTGTAA. The trimmed paired-end (PE) reads were mapped against the buckwheat reference sequence (FES_r1.0) using BWA 0.7.15 (Li and Durbin 2009) [42] with the -L 10 and -B 10 options. Single-end reads were filtered from BAM files using samtools 1.3.1. [43]. Variants were called by the samtools mpileup function and bcftools implemented in samtools 1.3.1. Sites with depths of less than 4 and more than 40 were converted to missing data using VCFtools 0.1.13 [44]. Then, sites with proportions of missing data greater than 0.2 were filtered using VCFtools 0.1.13.

### Construction of phylogenetic tree and structure analysis

ADMIXTURE v1.22 [45] was used to investigate the population structure of the 46 common buckwheat landraces. For each value of *K*, ten ADMIXTURE analysis runs were performed with different random seeds. The best run was selected according to the highest value of log likelihood. A neighbor-joining (NJ) tree [46] was constructed using SEQBOOT with 100 replicates, followed by the DNAdist (with the Kimura two-parameter method), Neighbor, and Consense programs from the PHYLIP package 3.6 [47]. The NJ tree was rooted using midpoint rooting and visualized with FigTree 1.4.2 [48]. Principal-component analysis (PCA) based on covariance was performed using Tassel 5.2.37 [49].

### The nucleotide diversity and F-statistics

Sites with a proportion of missing data greater than 0.2 were filtered using VCFtools 0.1.13. Then, the nucleotide diversity within species and each classification/group was calculated using VCFtools 0.1.13. Weir and Cockerham’s weighted *F*-statistics (Fst) [50] was calculated using VCFtools 0.1.13.

## Additional files

Additional file 1: **Figure S1.** Population structure of 46 accessions of buckwheat. A) Cross-validation errors of ancestral population assignment for different numbers of clusters by ADMIXTURE (K = 1–10). Mean cross-validation errors by 10 ADMIXTURE runs are shown with standard deviations. B) Population structure of 46 common buckwheat landraces inferred by ADMIXTURE (K = 2). Ancestry proportions for individuals were estimated using 255,517 SNPs. Color codes (cyan and magenta) of bars indicate typical genotypes of the inferred subpopulations. Red- and blue-colored accessions are short- and long-styled plants, respectively. (PNG 620 kb)

Additional file 2: **Figure S2.** Principal-component analysis (PCA) of 23 short-styled plants based on GBS data mapped on 332 S-allelic scaffolds. Graph of the first two axes (x-axis for PC1 and y-axis for PC2) from PCA is shown. The proportion of variance explained by each component is given in parentheses along each axis. (PNG 457 kb)

Additional file 3: **Table S1**. List of buckwheat landrcces used in this study. (PDF 51 kb)

## Abbreviations

AFLPs: Amplified fragment-length polymorphisms
GBS: Genotyping-by-sequencing
GBSS: Granule-bound starch synthases
PCA: Principal-component analysis
*S* locus: *Self-incompatibility* locus
S-ELF3: S-locus early flowering 3
SI: Self-incompatibility
SNPs: Simple nucleotide polymorphisms
SSG2: Short-style-specific gene 2
SSRs: simple sequence repeats (SSRs)
