# Supplementary figures and images for: Gene flow signature in the S-allele region of cultivated buckwheat
Source: BMC Plant Biol. 2019 Apr 3;19:125. doi: 10.1186/s12870-019-1730-1 (PMC6448236; doi:10.1186/s12870-019-1730-1)

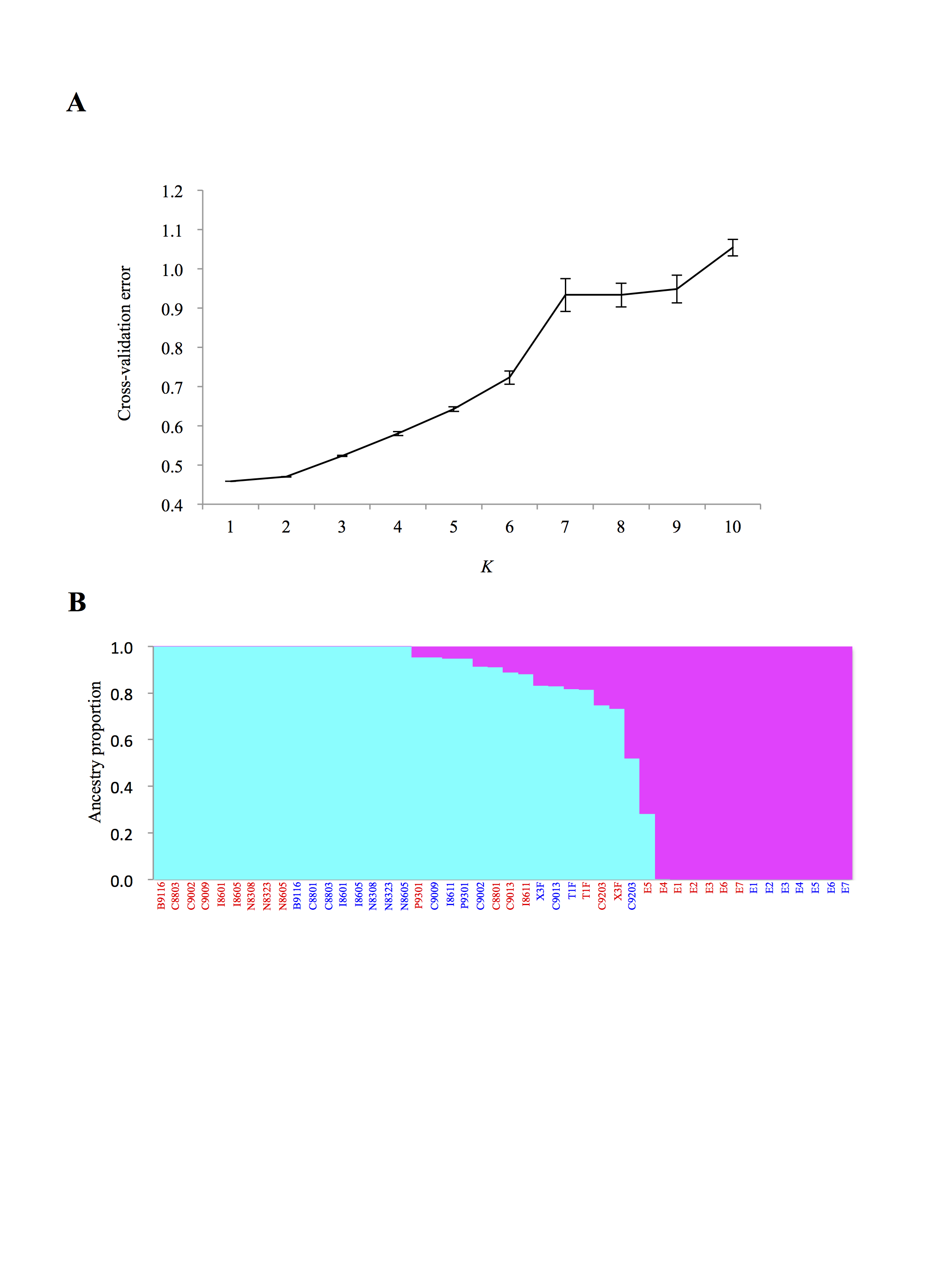

Supplement: Supplementary file 1 — Figure S1. Population structure of 46 accessions of buckwheat. A) Cross-validation errors of ancestral population assignment for different numbers of clusters by ADMIXTURE (K = 1–10). Mean cross-validation errors by 10 ADMIXTURE runs are shown with standard deviations. B) Population structure of 46 common buckwheat landraces inferred by ADMIXTURE (K = 2). Ancestry proportions for individuals were estimated using 255,517 SNPs. Color codes (cyan and magenta) of bars indicate typical genotypes of the inferred subpopulations. Red- and blue-colored accessions are short- and long-styled plants, respectively. (PNG 620 kb) [file 12870_2019_1730_MOESM1_ESM.png]

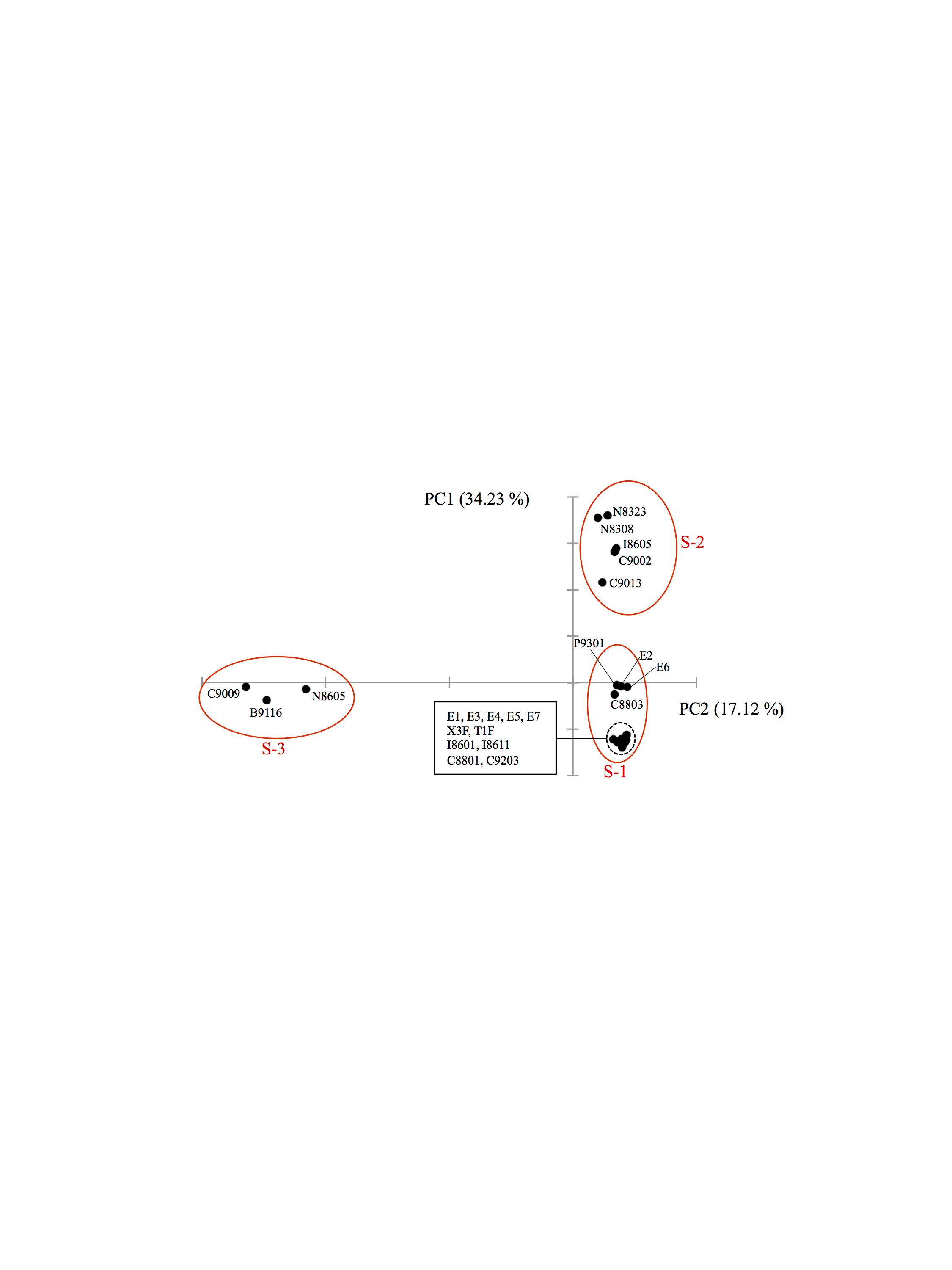

Supplement: Supplementary file 2 — Figure S2. Principal-component analysis (PCA) of 23 short-styled plants based on GBS data mapped on 332 S-allelic scaffolds. Graph of the first two axes (x-axis for PC1 and y-axis for PC2) from PCA is shown. The proportion of variance explained by each component is given in parentheses along each axis. (PNG 457 kb) [file 12870_2019_1730_MOESM2_ESM.png]
