# Supplementary material for: Gene flow signature in the S-allele region of cultivated buckwheat
Source: BMC Plant Biol. 2019 Apr 3;19:125. doi: 10.1186/s12870-019-1730-1 (PMC6448236; doi:10.1186/s12870-019-1730-1)
Supplement: Supplementary file 3 — Table S1. List of buckwheat landrcces used in this study. (PDF 51 kb) [file 12870_2019_1730_MOESM3_ESM.pdf]

Supplemantary Table S1. List of buckwheat landrcces used in this study.

Acession number[1]	Country	Flower type[2]	Number of reads (after trimming)	Total bases (after trimming)	Barcodes
B9116	Bhutan	short styled	5,008,172	505,825,372	AAACATC
C8801	China	short styled	9,409,428	950,352,228	AACGTGA
C8803	China	short styled	9,233,720	932,605,720	ACATTGG
C9002	China	short styled	6,145,192	620,664,392	ACCACTG
C9009	China	short styled	8,644,688	873,113,488	ACCTCCA
C9013	China	short styled	5,524,176	557,941,776	ACTATGC
C9203	China	short styled	8,200,012	828,201,212	AGAGTCA
I8601	India	short styled	5,862,146	592,076,746	AGCACCT
I8605	India	short styled	7,563,186	763,881,786	AGCAGGA
I8611	India	short styled	6,766,438	683,410,238	AGTGGTC
N8308	Nepal	short styled	6,965,884	703,554,284	ATCCTGT
N8323	Nepal	short styled	9,129,922	922,122,122	ATGCCTA
N8605	Nepal	short styled	7,774,788	785,253,588	ATTGAGG
P9301	Pakistan	short styled	7,268,822	734,151,022	CAAGGAG
T1F	Japan	short styled	7,605,966	768,202,566	CATCAAG
X3F	Japan	short styled	6,615,498	668,165,298	CCGAAGT
E1	France	short styled	5,707,240	576,431,240	CCTAATC
E2	Poland	short styled	7,401,406	747,542,006	CCTCCTG
E3	Russia	short styled	7,634,768	771,111,568	CGACACA
E4	Russia	short styled	6,601,016	666,702,616	CGCTGAT
E5	Slovenia	short styled	6,667,762	673,443,962	CGGATTG
E6	Slovenia	short styled	6,340,524	640,392,924	CTAAGGT
E7	Bosnia and Herzegovina	short styled	5,369,394	542,308,794	CTCAATG
B9116	Bhutan	long styled	6,992,938	706,286,738	CTGTAGC
C8801	China	long styled	7,376,990	745,075,990	GAACAGG
C8803	China	long styled	6,400,734	646,474,134	GAATCTG
C9002	China	long styled	7,040,138	711,053,938	GACTAGT
C9009	China	long styled	8,975,552	906,530,752	GAGCTGA
C9013	China	long styled	6,322,000	638,522,000	GATAGAC
C9203	China	long styled	7,137,374	720,874,774	GCCACAT
I8601	India	long styled	8,738,218	882,560,018	GCGAGTA
I8605	India	long styled	5,817,406	587,558,006	GGAGAAC
I8611	India	long styled	9,236,178	932,853,978	GGTGCGA
N8308	Nepal	long styled	5,891,144	595,005,544	GTACGCA
N8323	Nepal	long styled	8,158,326	823,990,926	GTCGTAG
N8605	Nepal	long styled	6,416,032	648,019,232	GTCTGTC

P9301	Pakistan	long styled	5,842,904	590,133,304	GTGTTCT
T1F	Japan	long styled	7,188,282	726,016,482	TATCAGC
X3F	Japan	long styled	6,476,654	654,142,054	TCCGTCT
E1	France	long styled	7,721,336	779,854,936	TCTTCAC
E2	Poland	long styled	8,172,300	825,402,300	TGAAGAG
E3	Russia	long styled	7,852,696	793,122,296	TGGAACA
E4	Russia	long styled	6,625,326	669,157,926	TGGCTTC
E5	Slovenia	long styled	7,782,752	786,057,952	TGGTGGT
E6	Slovenia	long styled	6,367,172	643,084,372	TTCACGC
E7	Bosnia and Herzegovina	long styled	8,722,206	880,942,806	TTGCAGA
Average			7,189,018	726,090,770	

[1] Samples from E1-E7 were obtained from European countries, and the other samples were

[2] Buckwheat is a heteromorphic self-incompatible species, and one accession contains both of
